# Mechanical Properties and Microstructural Characterization of Metakaolin Geopolymers Based on Orthogonal Tests

**DOI:** 10.3390/ma15082957

**Published:** 2022-04-18

**Authors:** Shoushuai Dai, Hongguang Wang, Shuai An, Long Yuan

**Affiliations:** School of Civil Engineering, Northeast Forestry University, Harbin 150040, China; daishoushuai@nefu.edu.cn (S.D.); anshuai@nefu.edu.cn (S.A.); 3352182709@nefu.edu.cn (L.Y.)

**Keywords:** geopolymer, metakaolin, mechanical properties, microstructure analysis

## Abstract

Metakaolin was used as a raw material for the preparation of geopolymers, where two types of alkali activators (Na_2_SiO_3_ + NaOH and Na_2_SiO_3_ + NaOH) were used to prepare metakaolin geopolymers at room temperature. The mechanical properties and microstructures of the metakaolin geopolymers were analyzed. A three-factor, four-level orthogonal test was designed to investigate the mechanical properties of the metakaolin geopolymer with different ratios. The compressive and flexural strength of different specimens were tested for 7 and 28 days. Both the Na-based and K-based geopolymers exhibited excellent mechanical properties, but the K-based geopolymer had better mechanical properties. The optimal compressive strength and flexural strength of the K-based geopolymer were 73.93 MPa and 9.37 MPa, respectively. The 28-day optimal compressive strength of the Na-based polymer was 65.79 MPa, and the flexural strength was 8.71 MPa. SEM, XRD, and FTIR analyses showed that the mechanical properties of the geopolymers could be greatly improved by using a higher alkaline solution concentration, proper Na_2_SiO_3_/MOH mass ratio, and proper mass ratio of alkali exciter to metakaolin. Amorphous silicoaluminate was more favorable for the dissolution of silicon–alumina raw materials, promoted the formation of an amorphous silicoaluminate gel, and caused the internal structure of the geopolymer to be more compact.

## 1. Introduction

In recent decades, with the development of the transportation and construction industries, the demand for cement has increased dramatically, and the production of cement has consumed a large amount of natural resources. Since the production of cement produces 5–7% of the world’s total CO_2_ gas emissions [1], finding alternatives to cement as a cementitious material for the construction industry has become an urgent problem. Geopolymers have excellent properties, such as good high-temperature stability [2], frost resistance [3], carbonation resistance [4], and acid resistance [5]. The production of geopolymers emits 80% less CO_2_ than ordinary silicate cement [6]; therefore, geopolymers are green cementitious materials with excellent properties and great potential for development. 

Since each researcher uses different raw materials and different curing methods, the mechanical strength of the prepared geopolymer is also quite different. Wan [7] synthesized geopolymers with quartz that was mechanically activated at different times and the strengthening mechanism of the geopolymers using activated quartz was investigated. With the activation time of quartz increasing from 0 to 60 min, the compressive strength of geopolymer increased from 31.5 to 55.2 MPa. D. E. Ortega-Zavala [8] studied the mechanical properties of metakaolin-based geopolymers using the Taguchi method with 12–16 wt.% R_2_O (R = Na or K). The experimental results showed that a geopolymer with a flexural strength of 19 MPa and compressive strength of 50 MPa could be obtained using uniaxial cold pressing at a low water–solid ratio and curing at 100–300 °C. P. N. Lemougna [9] investigated the effect of ground granulated blast furnace slag on the geopolymerization of low reactive volcanic ash. The results showed that the compressive strength increased with the addition of slag in the system until an optimal value of about 85 MPa. It can be seen that the geopolymer fully meets the strength standard of Portland cement and has excellent mechanical properties.

Since the discovery of geopolymers, besides using slag and fly ash as raw materials, many researchers have chosen to use metakaolin as a raw material for the preparation of geopolymers. Metakaolin contains more silica and alumina, has a lower impurity content and a stable chemical composition, and is purer compared to fly ash and slag [10]. In the current research, most researchers usually use the method of controlling a single variable to study geopolymers, keeping other influencing factors constant. This means that the whole test can only carry out the longitudinal comparison of individual factors and cannot carry out the overall analysis of each influencing factor. The lack of horizontal comparison of each influencing factor can lead to inefficient tests and the accuracy and comprehensiveness of the obtained test data can be adversely affected. Yunsheng et al. [11] proposed the first orthogonal experimental design method with three key factors, namely, SiO_2_/Al_2_O_3_, M_2_O/Al_2_O_3_, and H_2_O/M_2_O (M is Na or K), based on the chemical characteristics of metakaolin, to investigate the mechanical properties and microstructure of metakaolin geopolymers with nine different SiO_2_/Al_2_O_3_, Na_2_O/Al_2_O_3_, and H_2_O/Na_2_O compositions. The results showed that the Na_2_O/Al_2_O_3_ and H_2_O/Na_2_O ratios had significant effects on the compressive strength; the highest compressive strength was obtained with SiO_2_/Al_2_O_3_ = 5.5, Na_2_O/Al_2_O_3_ = 1.0, and H_2_O/Na_2_O = 7.0. Gaoshang Ouyang et al. [12] designed a three-factor, four-level orthogonal table to investigate the rheological and mechanical properties of metakaolin geopolymers. He noted that the fluidity of the geopolymers reached 144 mm and the compressive strength was optimal when SiO_2_/Al_2_O_3_ = 3.4, M_2_O/Al_2_O_3_ = 1, and H_2_O/M_2_O = 11. The orthogonal test method could reasonably arrange the testing, reduce the number of tests, and ensure that some possible scenarios are analyzed; furthermore, orthogonal test results are easy to analyze and can eliminate part of the interference caused by test errors.

An alkali activator can dissolve the silicon–aluminum raw material, provide an alkaline medium and metal cations, and is also an indispensable component to complete the geopolymerization reaction. At present, the commonly used alkali activators can be divided into two major categories, one using an alkaline solution or silicate solution as a single type of alkali activator (e.g., NaOH, KOH, Na_2_SiO_3_, K_2_SiO_3_) [13,14], while the other is a composite alkali activator in which the alkaline solution and silicate solution are mixed in a certain ratio [15,16]. The combined alkali activator has a better excitation effect than a single alkali activator and can prepare geopolymers with excellent mechanical properties [17]. Badr Aouan et al. [18] investigated the effect of different mix proportions of alkali activators on the mechanical properties of metakaolin geopolymer, with the main influencing parameters being the NaOH solution concentration, Na_2_SiO_3_/NaOH mass ratio, and solid–liquid mass ratio. The experimental results showed that when NaOH = 14 M, the Na_2_SiO_3_/NaOH mass ratio was 2.5, and the solid–liquid mass ratio was 1.5, a large number of amorphous aluminosilicate gels were formed in the specimens, which improved the compressive strength of the specimens, with the highest strength being 34.81 MPa. Hsiao Yun Leong et al. [19] investigated the effect of alkali activator types on the compressive strength of fly ash geopolymer. The results showed that the highest compressive strength was achieved when Na_2_SiO_3_/NaOH = 2, Na_2_SiO_3_/KOH = 1, and the alkali activator/ash ratio was 0.4, and as the Na_2_SiO_3_ content in the samples increased, more Si content was provided to the reaction system, which contributed to the increase in compressive strength. It was concluded that the use of composite alkali activators can have a super-stacking effect to obtain geopolymers with better mechanical properties.

In this test, two types of alkali activators (Na_2_SiO_3_ + NaOH and Na_2_SiO_3_ + KOH) were used to prepare the geopolymers at room temperature, with metakaolin as the raw material. The optimal mixing ratio of geopolymers was determined through the compression and bending test to ensure that its mechanical properties met the requirements of road repair, grouting materials, and other related projects. In this experiment, three key factors (alkaline solution concentration, alkali-activator-to-metakaolin mass ratio, and Na_2_SiO_3_/MOH mass ratio) were selected to investigate the mechanical properties of metakaolin geopolymers. The microscopic mechanism analyses were performed using SEM, XRD, and FTIR. The microscopic mechanisms of the interactions between different factors were given according to the microscopic morphology, the composition of the physical phases, and the functional groups contained in the different specimens, and the differences in the mechanical properties of the specimens were reasonably explained.

## 2. Experimental Materials and Methods

### 2.1. Materials

#### 2.1.1. Metakonlin (MK)

**Figure 1:**
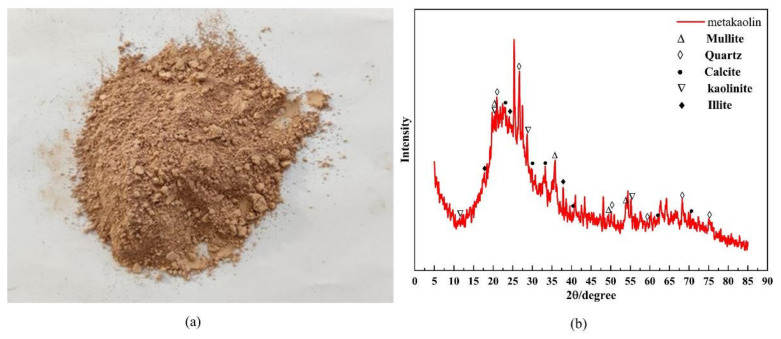
Raw materials and phase composition: (**a**) metakaolin and (**b**) XRD analysis of metakaolin.

The raw material used in this research was highly reactive metakaolin produced by Gongyi Jin’ao Resistance Material Co., Ltd. (Gongyi, China, with a fineness of 1250 mesh (Figure 1a). The chemical composition and mass fraction were determined using X-ray fluorescence spectroscopy analysis and the specific results are shown in Table 1. Among them, SiO_2_ and Al_2_O_3_ accounted for 89% of the chemical composition of the metakaolin; CaO accounted for 3.88%; and other substances, such as Na_2_O, K_2_O, and Ti_2_O, accounted for 5.83%. A physical phase analysis (XRD) of metakaolin was carried out, and the specific results are shown in Figure 1b. The metakaolin contained more quartz phase, calcite phase, and kaolinite phase, and had a broad diffraction peak at 18°–30° (2*θ*), indicating that it contained many amorphous silicoaluminate minerals [6], which is a highly reactive mineral admixture.

#### 2.1.2. Alkali Activator

There were two types of alkali activators used in this experiment: the first one was sodium hydroxide solution mixed with sodium silicate solution in a certain ratio (NaOH + Na_2_SiO_3_) and the second one was potassium hydroxide solution mixed with sodium silicate solution in a certain ratio (KOH + Na_2_SiO_3_). The solid sodium hydroxide or potassium hydroxide required for the test was weighed in a beaker. Water was added to the beaker and was continuously stirred with a glass rod to prepare an alkaline solution of the desired concentration. For example, to configure 1 L of NaOH solution (14 mol/L), 583.3 g of NaOH solid was weighed in a beaker and water was added to make the solution volume equal to 1000 mL. Then, according to the orthogonal table, the alkaline solution and the sodium silicate solution were mixed in a certain mass ratio to prepare an alkaline activator. Finally, the prepared alkali activator was sealed with a plastic film and left to stand. Technical index: the solid content of sodium silicate solution was 34.2% (26% SiO_2_, 8.2% Na_2_O), °Bé was 38, and the modulus was 3.2. The sodium hydroxide was a white granular substance with 96% analytical purity, while the potassium hydroxide was a white flaky solid with 85% analytical purity.

### 2.2. Orthogonal Experimental Design

In this experiment, the mechanical properties of the metakaolin geopolymer were investigated using a three-factor, four-level orthogonal test table. Factor A was the concentration of the alkaline solution, factor B was the mass ratio of alkali exciter to metakaolin, and factor C was the mass ratio of sodium silicate solution to alkaline solution (NaOH solution or KOH solution). The alkaline solution concentrations were 8 mol/L, 10 mol/L, 12 mol/L, and 14 mol/L; the mass ratios of the alkali activator to metakaolin were 0.7, 0.8, 0.9, and 1.0; and the mass ratios of the sodium silicate solution to alkaline solution were 1.5, 2.0, 2.5, and 3.0. The compressive and flexural strengths of the specimens at 7 and 28 days were used as a measure of the mechanical properties. For the convenience of presentation, the geopolymer excited using Na_2_SiO_3_ + NaOH was referred to as the Na-based geopolymer and the geopolymer excited using Na_2_SiO_3_ + KOH was referred to as the K-based geopolymer. The orthogonal table used in this experiment is shown in Table 2.

### 2.3. Specimen Preparation

A JJ-5 planetary mixer was used for the preparation of specimens. Before mixing, the metakaolin was prepared by pouring it into the mixing pot according to the design ratio. Then, the prepared alkali activator was slowly poured into the mixing pot, the planetary mixer was started, and the mixture was mixed slowly for 1 min and then mixed quickly for 2 min to cause it to form a uniform geopolymer slurry. The slurry was prepared by pouring it into 40 mm × 40 mm × 40 mm and 40 mm × 40 mm × 160 mm triplex molds and vibrated fully for 1 min to remove the air bubbles in the specimen after the pouring was completed. The specimens were maintained in the laboratory using normal temperature maintenance and covered with cling film to prevent cracks from water loss. After curing for 24 h, the specimens were demolded and continued to be maintained in the laboratory environment until they were fully aged and tested for mechanical properties.

### 2.4. Test Methods

#### 2.4.1. Mechanical Properties Testing

**Figure 2:**
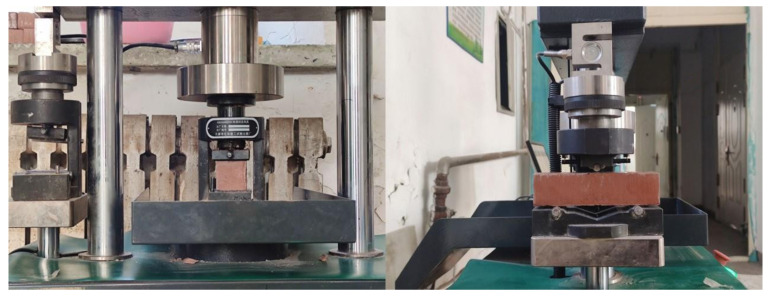
Mechanical properties test chart of a sample.

The specific test operation is shown in Figure 2. 

According to the national specification GB/T 17671-1999 [20], a comprehensive mechanical testing machine was used to conduct the compressive and flexural strength tests. When conducting the flexural strength test, the specimen size was 40 mm × 40 mm × 160 mm, the three-point loading method was used, and the loading rate of the testing machine was 50 N/s. When the specimen was broken, the test data were recorded. In the compressive strength test, 40 mm × 40 mm × 40 mm cubic specimens were used for testing and the loading rate was 2.4 kN/s. When the specimens were damaged by compression, the peak data was recorded as the compressive strength of this test. In a set of mechanical property tests, the mechanical strength of three specimens was tested to find the average value to reduce the test error and ensure the accuracy of the results. 

#### 2.4.2. Microscopic Mechanism Analysis

To better determine the effects of different mix proportions on the mechanical properties of metakaolin geopolymer, microscopic mechanism analyses, including scanning electron microscopy (SEM), X-ray diffraction analysis (XRD), and Fourier transform infrared (FTIR) spectroscopy, were performed on the geopolymer specimens prepared with two types of alkali activator, and the age of the tested specimens was 28 days. X, Pert3 Powedr*, and */XRD-6100 model X-ray diffractometers were used for scanning in the ranges of 5–85° and 5–90° with scanning steps of 0.0065651 and 0.02, respectively. FTIR was performed using the ART method with a Nicolet iN10/Fourier transform microinfrared spectrometer. The microstructure of the specimens was observed using an EM-30 plus benchtop SEM at accelerating voltages of 1~30 kV with magnifications of 2000× and 5000×.

### 2.5. Polar Difference Methodology

In the current research, most researchers usually use the method of controlling a single variable to study geopolymers, keeping other influencing factors constant. This means that the whole test can only carry out the longitudinal comparison of individual factors and cannot carry out the overall analysis of each influencing factor. The lack of horizontal comparison of each influencing factor can lead to inefficient tests, and the accuracy and comprehensiveness of the obtained test data can be adversely affected. The polar difference methodology can better explore the influence of various factors on the mechanical properties of geopolymers and comprehensively analyze the changing trend of mechanical properties, which is helpful for finding the optimal mix ratio of geopolymers. Here, we used a three-factor, four-level orthogonal test that was designed with three key factors (concentration of alkaline solution, mass ratio of alkali activator to metakaolin, and mass ratio of sodium silicate solution to alkaline solution) to investigate the mechanical properties of metakaolin geopolymer at different ratios.

## 3. Results and Discussion

### 3.1. Mechanical Properties Analysis

The mechanical properties of the metakaolin geopolymers were analyzed in orthogonal tests, where the specific experimental arrangements and test data are shown in Table 3 and Table 4. From the experimental data, it could be seen that different factors had different degrees of influence on the mechanical properties of the geopolymers, and there were differences in the mechanical strengths of the geopolymers prepared with different types of alkali activators. For the Na-based geopolymer, the maximum compressive strength of the test group at 28 days was 64.18 MPa and the flexural strength was 8.28 MPa. For the K-based geopolymer, the maximum compressive strength of the test group at 28 days was 72.72 MPa and the maximum flexural strength was 8.66 MPa. He [21] prepared metakaolin-based geopolymers using a controlled single-variable method. The highest compressive strength at 7 days was 50.21 MPa and the highest flexural strength was 7.66 MPa. Liang [22] prepared geopolymers by incorporating rice husk ash and silica fume into metakaolin. When the content of rice husk ash was 20 wt.% or the content of silica fume was 10 wt.%, the highest compressive strength was obtained and the compressive strengths at 28 days were 56.5 MPa and 48.4 MPa, respectively. It can be seen that the orthogonal test designed in this study could analyze various influencing factors more comprehensively, and prepare geopolymers with better mechanical properties. Overall, the mechanical properties of the K-based geopolymer were better than those of the Na-based geopolymer.

In this experiment, polar difference analysis was used to analyze and integrate the experimental data and calculate the K and k values of the geopolymers for each factor and at different levels. We thus obtained the polar difference R of each factor at different levels and then compared the primary and secondary order of the influence of each factor on the mechanical properties according to the magnitude of the R value. The optimal level and the best combination of each influencing factor were determined by comparing the k values. In this experiment, the K value was the sum of four sets of test data for each factor at the same level, and the k value was the average of four sets of test results for each factor at the same level. The specific data are shown in Table 5, Table 6, Table 7 and Table 8.

From Table 5 and Table 6, it can be seen that for the Na-based geopolymers, different factors had different degrees of influence on their mechanical properties. For the compressive strength, the magnitudes of the R values for both 7 days and 28 days were in the order of R_A_ > R_B_ > R_C_, indicating that the alkaline solution concentration’s had the greatest effect on the compressive strength of the Na-based geopolymers and Na_2_SiO_3_/NaOH had the least effect on the compressive strength. By comparing the k values of the four levels of factor A, we concluded that k_4_ > k_3_ > k_2_ > k_1_, i.e., the optimal level of factor A was level 4. Similarly, the optimal level of factor B was level 3 and the optimal level of factor C was level 1. Therefore, the optimal ratio to obtain the best compressive strength of the metakaolin geopolymer was A_4_B_3_C_1_. The concentration of NaOH solution was 14 mol/L, the (Na_2_SiO_3_ + NaOH)/MK mass ratio was 0.9, and the Na_2_SiO_3_/NaOH mass ratio was 1.5. By analogy, it was concluded that the optimal ratio of flexural strength of the Na-based geopolymer was A_4_B_3_C_2_, the optimal ratio of compressive strength of the K-based geopolymer was A_3_B_2_C_4_, and the optimal ratio of flexural strength of the K-based geopolymer was A_4_B_1_C_3_. The optimal compressive strength of the Na-based geopolymers and the optimal flexural strength of the K-based geopolymers were not included in the test group from the extreme difference analysis. After subsequent tests, the 7-day optimal compressive strength of the Na-based geopolymer was 61.97 MPa and the 28-day optimal compressive strength was 65.79 MPa. The 7-day optimal flexural strength of the K-based geopolymer was 8.63 MPa and the 28-day optimal flexural strength was 9.37 MPa.

### 3.2. Analysis of Influencing Factors

**Figure 3:**
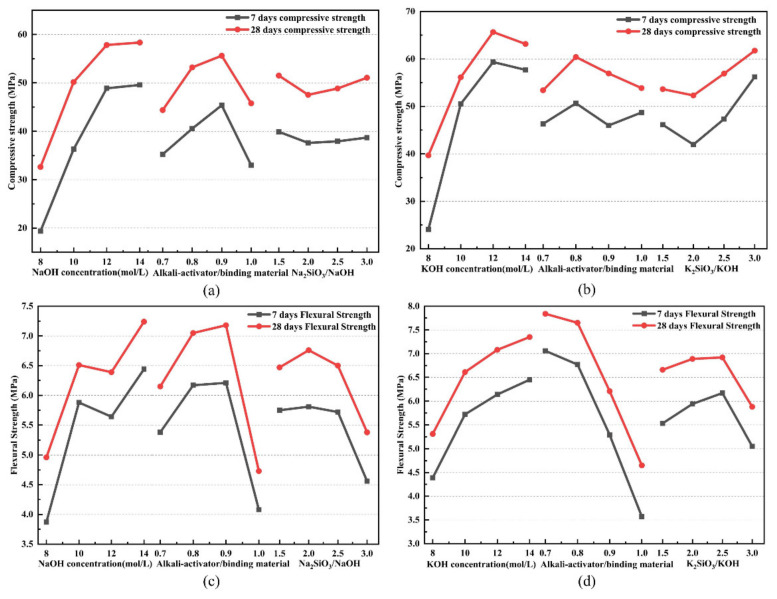
Factorial diagrams of the main parameters: (**a**) factorial diagrams of the main parameters that affected the compressive strength of the Na-based geopolymer samples, (**b**) factorial diagrams of the main parameters that affected the compressive strength of the K-based geopolymer samples, (**c**) factorial diagrams of the main parameters that affected the flexural strength of the Na-based geopolymer samples, and (**d**) factorial diagrams of the main parameters that affected the flexural strength of the K-based geopolymer samples.

The primary and secondary relationships of the effects of each factor on the mechanical properties of the Na-based and K-based geopolymers were obtained using polar difference analysis. The trend graphs of the effects of each factor on the mechanical properties were plotted to specifically analyze the variation patterns of the effects of the alkaline solution concentration, mass ratio of alkali activator to metakaolin, and mass ratio of Na_2_SiO_3_/MOH on the mechanical properties for further in-depth study. The specific trend diagrams are shown in Figure 3.

It can be seen that for the Na-based and K-based geopolymers, the influence patterns of each factor on their compressive strengths were similar. The alkaline solution concentration had the greatest effect on the compressive strength of both geopolymers. For the Na-based geopolymers, the effect of alkaline solution concentration on the compressive strength was positively correlated, while for the K-based geopolymers, it showed an increasing and then decreasing trend. The compressive strength of the geopolymer increased with increasing curing time at higher NaOH concentrations, and the rise in alkali concentration increased the dissolution of silicon and aluminum in the raw material, resulting in the formation of more silicate and aluminate monomers in the reaction system, causing the reaction to be more complete and thus increasing the compressive strength of the geopolymer [23]. The compressive strength of the K-based geopolymers was higher than that of the Na-based geopolymers due to the fact that the K^+^ in the KOH solution facilitated the formation of ion-pair (cation–anion) reactions and generated larger silicate oligomers; furthermore, the presence of such geopolymer precursors (long-chain silicate oligomers and Al-O-Si pairs) increased the compressive strength [19]. The KOH solution is more alkaline compared to the NaOH solution, which leads to a significant increase in the dissolution and polymerization rate of raw materials containing silicon–aluminum [24]. If the concentration of KOH is too high, it will cause the geopolymerization reaction to be too violent, and some dissolved silicon–aluminum monomers are often wrapped by the just-formed coalescence before the reaction occurs, which will make their structure less dense and lead to a decrease in compressive strength. As the mass ratio of Na_2_SiO_3_/MOH increased, the compressive strength of both geopolymers first decreased and then increased. When the Si content in the system was low, the condensation process mainly formed oligomeric silicates with (-Si-O-Al-O-) polymer structures, and when the Si content increased, 3D structures of (Si-O-Al-O-Si-O) and (Si-O-Al-O-Si-O-Si-O) were formed, and they possessed higher stability and rigidity than the (-Si-O-Al-O-) structures, resulting in structures with higher strength [25]. With the increase in the mass ratio of alkali activator solution to metakaolin, the compressive strength of both geopolymers showed a tendency to rise first and then fall. When the liquid content in the system increases, the silicon–aluminum structure in metakaolin is more easily disintegrated by the alkali ions in the liquid, which increases the solubility of the raw material and promotes the migration of ions in the slurry, forming more amorphous silicoaluminate gels, causing the structure of the geopolymer to be denser and increase the compressive strength. However, if the liquid content is too large, the excess water in the system does not participate in the geopolymerization reaction, and after curing is completed, pores are created inside the structure due to the evaporation of water, which adversely affects the mechanical properties [26,27].

The patterns of influence of the three factors on the flexural strength of the Na-based and K-based geopolymers were different from that of the compressive strength. The flexural strength of both geopolymers showed an increasing trend as the concentration of the alkaline solution increased. This can be explained due to the fact that as the alkaline solution concentration increases, the pH value of the alkali activator increases, the alkalinity is enhanced, and there is more OH^−^ in the system, which causes the amorphous Si-O and Al-O bonds on the surface of metakaolin to be more easily broken and more oligomers of silica and aluminate are formed after destruction. As more oligomers are formed, further geopolycondensation reactions occur and polymerize into ionic clusters, which eventually harden into a three-dimensional network-like structure and improve the mechanical properties of the geopolymers [28]. The sodium silicate solution generally acts as a binder in the geopolymerization reaction, and with the increase in the mass ratio of Na_2_SiO_3_/NaOH, the specimen reaches the maximum flexural strength when there is a sufficient amount of alkali activator, but with the increase in the mass ratio, excess silicate will precipitate, which is not conducive to the dissolution of the silicon–aluminum raw material and destroys the geopolymerization reaction, resulting in a decrease in strength [29]. Moreover, if the mass ratio of alkali activator to metakaolin is too large, it will adversely affect the flexural strength of the geopolymer. As the mass of the alkali activator solution increases, the water-to-solid ratio in the system also increases, and under high water-to-solid ratio conditions, the concentration of the alkaline solution is diluted, making the reaction drive insufficient and leading to a decrease in flexural strength [30]. When the mass ratio of alkali activator to metakaolin is reduced, the solids content increases and the contact between the activating solution and the reacting material is improved, which can promote the dissolution of the silicon–aluminum structure in the raw material and improve the mechanical properties of the geopolymer [31].

### 3.3. Microscopic Mechanism Analysis

The macroscopic mechanical properties of geopolymers can be analyzed and verified from a microscopic mechanism point of view. In order to better explain the causes of the differences in the mechanical properties of the geopolymers, the corresponding specimens were selected from the available test groups for microscopic mechanism analysis: N_1_, N_2_, N_7_, and N_14_ were selected from Table 3 and K_2_, K_3_, K_5_, K_7_, K_9_, and K_15_ were selected from Table 4 for microscopic mechanism tests. The basis for this selection was to ensure that the level of one of the three factors was optimal, while the levels of the other two factors were not at the optimal level, which could better reflect the effect of different factors and different levels on the microstructure of the geopolymer. The advantages and disadvantages of the mechanical properties of the geopolymers were analyzed in detail via XRD, SEM, and FTIR analysis from the perspective of microscopic reaction mechanisms.

#### 3.3.1. XRD Analysis

**Figure 4:**
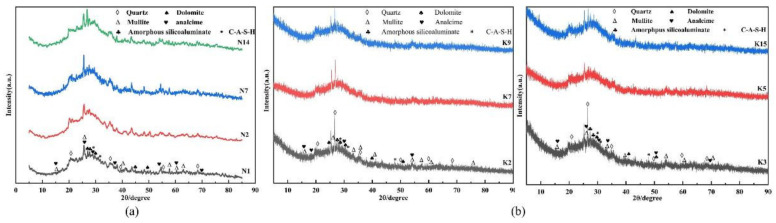
The 28-day XRD patterns of geopolymers: (**a**) XRD pattern of Na-based geopolymers and (**b**) XRD pattern of K-based geopolymers.

The XRD patterns of both the Na-based and K-based geopolymers at 28 days were plotted separately, as shown in Figure 4a,b.

In metakaolin, kaolinite is used as the main mineral component, and the characteristic peak 2*θ* values of kaolinite were mainly 12.3°, 19.8°, 24.9°, and 45.4°, in addition to quartz, mullite, calcite, and other minerals. After the alkali excitation reaction, the reflection peaks of these substances were still present in the system, which indicated that they were mostly inactive and basically did not participate in the geopolymerization process [31]. Combining with Figure 4a,b, it can be obtained that the results of the geopolymerization mainly showed the typical variation of the amorphous broad hump, the position of the amorphous broad hump was between 20° and 36°, and the center of the Na-based geopolymer and K-based geopolymer humps gradually moved toward 27.4° and 27.8°, respectively. This indicated that some of the amorphous phase material in the metakaolin was dissolved by the alkali activator and a new amorphous phase material is formed. As can be seen in Figure 4, although the Na-based and K-based geopolymers still had quartz, mullite, and dolomite phases after 28 days of curing, the peaks of these phases decreased with increasing alkaline solution concentrations. Compared with the XRD of metakaolin, their peak intensities were significantly weaker at 26°–30° (2*θ*), indicating that the dissolution of silicon–aluminum raw materials in the metakaolin was promoted with increasing alkaline solution concentration [30], releasing more silicon–aluminum monomers and favoring the formation of silicoaluminate gels. The hydrated silicoaluminate gel is the main reaction product in the alkali activation reaction and it is mainly responsible for the development of the strength of the geopolymer. In addition, metakaolin contains a small amount of CaO, which is involved in the reaction after dissolution, and diffraction peaks of hydrated calcium silicoaluminate gels (C-A-S-H) were also detected in the geopolymer [32]. Due to the amorphous and semi-amorphous properties of hydrated silicoaluminate gels and C-A-S-H gels, which are often described as “featureless peaks” and difficult to index, these phases in the geopolymer form broad diffraction peaks at 2*θ* of 28–30° and 34–35° [33]. Comparing the XRD patterns of N_1_, N_2_, and N_14_, and those of K_2_, K_7_, and K_9_, it can be seen that the diffraction peak intensity of the silicoaluminate gels produced by the alkali excitation reaction gradually increased and the diffraction peak shifted to the right as the concentration of the alkaline solution increased and the mass ratio of Na_2_SiO_3_/MOH increased. This increase in peak intensity and the rightward shift of the diffraction peak was associated with a higher degree of geopolymerization reaction, producing more gels to fill the voids in the structure, resulting in improved mechanical properties of the geopolymer. When the alkali activator is mixed with metakaolin, the OH^−^ in the alkali activator breaks the Al-O, Si-O, Al-O-Al, Al-O-Si, and Si-O-Si bonds in the silica-rich and aluminum-rich phases, releasing Al(OH)_4_^−^ monomers and Si(OH)_4_ monomers. The Si(OH)_4_ monomer reacts with Ca^2+^ to form a C-S-H gel, and part of the Al goes into the C-S-H gel in place of Si, forming a C-A-S-H gel [34]. In addition, Al(OH)_4_^−^ monomers and Si(OH)_4_ monomers combine with alkali metal cations to form hydrated silicoaluminate gels or zeolite phase products [35]. Thus, the amorphous peaks of the metakaolin geopolymers correspond to a composite of C-S-H, C-A-S-H, and hydrated silicoaluminate gel phases that intertwine and together improve the mechanical properties of geopolymers. In Figure 4, it can be observed that some zeolite phases were still present in both the Na-based and K-based geopolymers. It was claimed that Na^+^ has better zeolitization ability in the geopolymer system due to its smaller size and easier migration through the gel network than K^+^ [24], but the system with more zeolite products formed tends to make the geopolymer less strong [23]. It is one of the reasons why Na-based geopolymers are slightly weaker than K-based geopolymers in terms of mechanical properties.

#### 3.3.2. SEM Analysis

**Figure 5:**
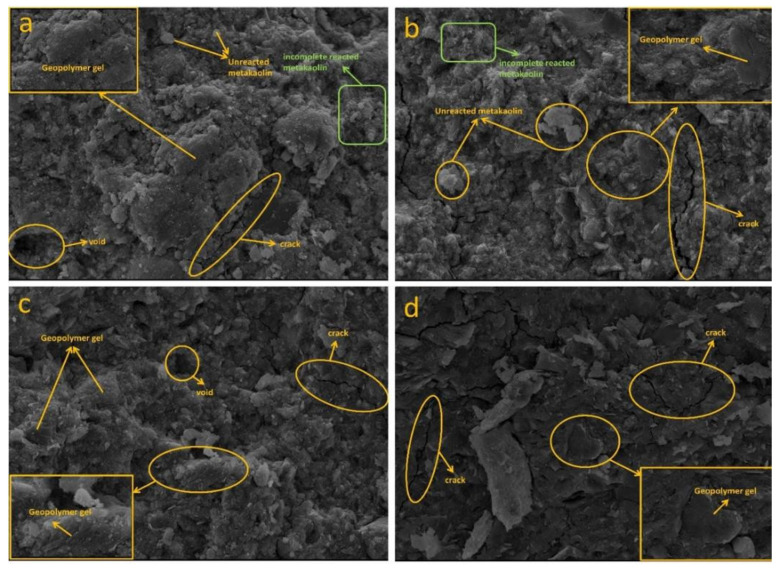
The 28-day SEM microscopic morphology of Na-based geopolymers (N1, N2, N7, and N14 in (**a**–**d**), respectively).

**Figure 6:**
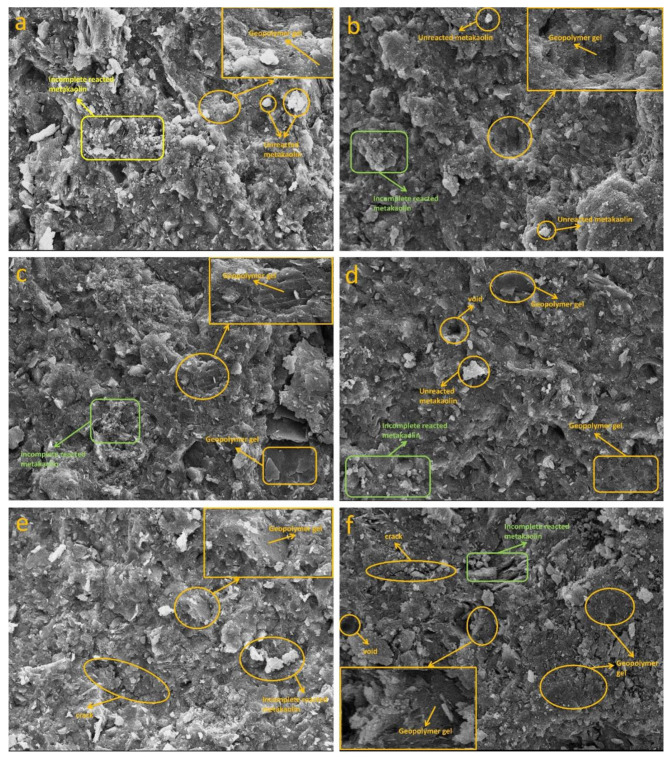
The 28-day SEM micromorphology of K-based geopolymers (K2, K3, K5, K7, K9, and K15 in (**a**–**f**), respectively).

The SEM microscopic images of Na-based and K-based geopolymers cured at room temperature for 28 days are shown in Figure 5 and Figure 6. Figure 5a shows that when the alkaline solution concentration was low, there was a considerable amount of incompletely reacted metakaolin in the N_1_ specimens. At the same time, the microstructure of the specimens also showed that there were some microcracks and pores in the geopolymer matrix, which were responsible for the poor mechanical properties of N_1_ in the test group. The concentration of the alkaline solution was too low to dissolve sufficient silicon–aluminum monomers, resulting in a large number of unreacted or incompletely reacted metakaolin particles present in the specimen matrix. This becomes a structural defect in the matrix and hinders the development of the gel phase network in the geopolymer, degrading the mechanical properties of the geopolymer [29,36]. Yuan’s research [37] showed that the presence of more microcracks in the specimens indicates that there is a delay in the geopolymerization reaction, which may be due to the slow activation of the early reaction system with low alkali content or fast early activation. The unreacted metakaolin particles are wrapped in silicoaluminate gel, which hinders their chemical reaction with the alkali activator. With the increase in the alkali solution concentration, it can be seen from Figure 5c,d that the unreacted metakaolin decreased and a gradually denser gel phase presented in the matrix, which caused the internal structure of the geopolymer to become denser. According to the XRD analysis, it is clear that the coexistence of N-A-S-H and C-(A)-S-H in the gel system filled the voids present in the structure and combined adjacent solids together to form a continuous and dense matrix structure [38], which improved the mechanical properties of the specimens. By comparing Figure 5a,b, it can be seen that the microstructure of the geopolymer in (b) was a little denser and more gels were produced. Specimen N_2_ contained more sodium silicate solution than specimen N_1_, where the use of silicate helped to enhance the geopolymerization process, accelerated the dissolution of silica and alumina in metakaolin, and increased the sodium ion content of the mixture, which acted as a charge balancing ion that promoted the formation of silicoaluminate gels [18]. This further confirmed that an appropriate increase in the mass ratio of Na_2_SiO_3_/NaOH is beneficial to the formation of the geopolymer gel and the development of the strength of the specimen. In the K-based geopolymers, there was also a pattern of strength development as previously described, with increasing concentration of alkaline solution, increasing mass ratio of Na_2_SiO_3_/KOH, and a gradual increase in the alkali activator content, where more gel material is observed to be generated in the reaction system, which resulted in a denser matrix structure. Figure 5c and Figure 6d show the microstructures of the two geopolymers at the same mix proportion (alkaline solution concentration of 10 mol/L, alkali activator to metakaolin mass ratio of 0.9, and Na_2_SiO_3_/MOH mass ratio of 3). By comparing the microscopic images of the Na-based and K-based geopolymers, it is obvious that the K-based geopolymers had much fewer microcracks and voids than the Na-based geopolymers and the microstructure of K-based geopolymer was denser. The difference between the two geopolymers was that the alkaline solution types were different and their alkali activating ions were Na^+^ and K^+^, respectively. Since the atomic number of K is greater than Na, the alkalinity of the KOH solution is greater than NaOH solution at the same concentration. The greater the alkalinity of the reaction system, the faster and fuller the dissolution of the silicon–aluminum raw material, which caused the K-based reaction system to have a higher concentration of reactive groups and the geopolymerization reaction was more complete [39].

In fact, no significant differences in the appearance of the prepared geopolymers were observed, regardless of whether KOH or NaOH was used as the alkaline solution. More unreacted metakaolin particles, as well as more microcracks, could be observed in the Na-based geopolymers, whereas in the K-based geopolymers, most of them appeared as homogeneous dense gels, which was strongly related to the nature of the alkaline solution. The size of Na^+^ is smaller than K^+^, and Na^+^ prefers to form pairings with smaller silicate oligomers, such as silicate monomers. The paired form is not conducive to binding to another silicate anion and does not readily form larger silicate oligomers. In contrast, the larger size of K^+^ is more conducive to the formation of larger silicate oligomers; therefore, systems using KOH solutions containing more geopolymer precursors [40] can fill the voids present in the matrix, giving the geopolymer better adhesion and not affecting the development of mechanical properties due to the presence of too many internal pores and microcracks.

#### 3.3.3. FTIR Analysis

**Figure 7:**
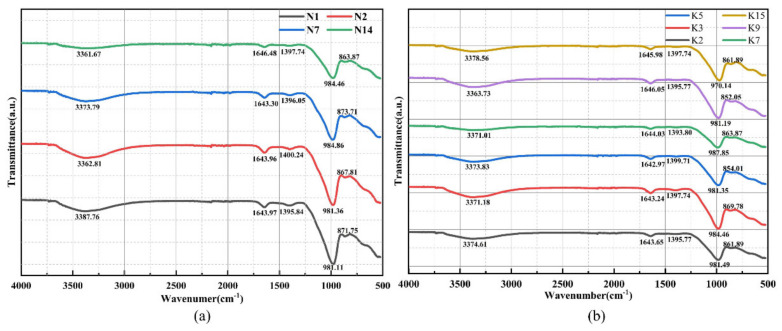
The 28-day FTIR spectra of geopolymers: (**a**) FTIR spectrum of Na−based geopolymers and (**b**) FTIR spectra of K−based geopolymers.

FTIR analysis was performed for the Na-based and K-based geopolymers with different mix proportions; the FTIR patterns of the two geopolymers at 28 days are shown in Figure 7a,b.

The FTIR technique for the detection of geopolymers can be used to more effectively analyze the bonding states of Si-O and Al-O and the changes in the coordination environment in silicon–aluminum raw materials [41]. Generally, FTIR spectra can be divided into two main spectral regions: the low-frequency region of 400–1400 cm^−1^ and the high-frequency region of 1400–4000 cm^−1^ [42,43]. From Figure 7a,b, it can be seen that all geopolymer specimens had a strong absorption peak at 970–980 cm^−1^ for the asymmetric stretching vibration of Si-O-T (T = Si or Al), formed by (AlO_4_)_4_^−^ replacing (SiO_4_)_4_^−^, where Al^3+^ replaces Si^4+^ in Si-O-Si, forming an aluminum–oxygen tetrahedral structure. It causes a change in the internal structure of the system, which indicates the generation of aluminosilicate gels in the geopolymer. These FTIR bands corresponded to the abundance of quartz and illite in the studied materials. Judging by the sizes and positions of the 970–980 cm^−1^ FTIR bands, the quantity of quartz and clay minerals decreased successively in the N_1_, N_2_, N_7_, and N14 and K_3_, K_5_, and K_9_ materials [44]. The higher the height of the characteristic band, the less raw material was depolymerized in the reaction system. This caused the geopolymerization reaction to be not complete enough and had a bad influence on the mechanical properties of the specimen. This argument also corresponds to the mechanical properties of the specimens in Section 3.1. In Figure 7b, as the KOH solution can increase the content of Al in the system, the vibrational energy contained in Al-O is lower than that of Si-O; therefore, the Si-O-T vibrational peak will move to the lower wavenumber range in the K-based geopolymer [45,46]. The geopolymerization reaction is exothermic and the energy of the whole system will gradually decrease as the reaction proceeds, and the more thoroughly the geopolymerization reaction proceeds, the lower the Si-O-T wave number will be [12]. Comparing N_1_, N_2_, and N_14_ in Figure 7a and K_3_, K_7_, and K_15_ in Figure 7b, it can be seen that the Si-O-T absorption peaks of both geopolymers shifted toward the low wavenumber range when the influencing factors reached the optimal level or the suboptimal level. It further verified that increasing the alkaline solution concentration and using appropriate (Na_2_SiO_3_ + MOH)/MOH and Na_2_SiO_3_/MOH mass ratios could promote the geopolymerization reaction and the formation of more silicoaluminate gels. In addition, the characteristic peaks at 3361–3387 cm^−1^ and 1642.97–1646.48 cm^−1^ in Figure 7 corresponded to the absorption bands of water molecules. The former is related to the presence of structural and free water in the geopolymer matrix and corresponds to the stretching vibration of [OH]^−^, while the latter corresponds to the bending vibration of H-O-H [30]. Apparently, these bands show the absorption of closed water in the sample cavities or water from the surface of the geopolymer structure [47]. Via longitudinal comparison, in Figure 7a,b, it can be seen that the intensity of the characteristic peak related to water molecules gradually decreased and finally almost disappeared with the increasing concentration of the alkaline solution. This indicated that with the increasing concentration of the alkaline solution, the reaction water was lost more quickly within the reaction system, making the matrix structure denser and improving the mechanical properties. In the Na-based and K-based geopolymers, the absorption peaks at 1393.80–1400.24 cm^−1^ and 852.05–873.71 cm^−1^ corresponded to the stretching vibration of CO_3_^2−^. The former corresponds to the asymmetric stretching vibration of O-C-O and the latter is due to the out-of-plane bending vibrations [46]. This indicates that carbon dioxide in the air reacts with the remaining alkali in the geopolymer, resulting in carbonation of the structure itself to form carbonates or bicarbonates, leading to a decrease in the strength of the geopolymer. By analyzing the patterns of N_2_ and K_2_, as well as N_7_ and K_7_, the vibrational peak intensities of the K-based geopolymer were weaker than the Na-based geopolymers at the same mix proportion. This indicated that the geopolymerization reaction of a K-based geopolymer was more complete, forming a denser gel structure that was less affected by carbonation, further verifying that a K-based geopolymer has better mechanical properties.

## 4. Conclusions

In this study, metakaolin geopolymers were prepared at room temperature. The effects of the alkaline solution concentration, mass ratio of alkali activator to metakaolin, and Na_2_SiO_3_/MOH mass ratio on the mechanical properties of metakaolin-based geopolymers were investigated using orthogonal tests using a three-factor, four-level orthogonal table. Based on the experimental results, the following conclusions could be drawn.

(1) Both the Na-based and K-based geopolymers could achieve high compressive and flexural strengths. They all had early-strength properties, with mechanical strengths of up to 80% of the 28-day strength at 7 days, but the mechanical properties of the K-based geopolymers were better than those of the Na-based geopolymers.
(2) For the compressive strength, the increase in alkaline solution concentration increased the compressive strength of the geopolymers, and the Na-based geopolymers always maintained a positive relationship, while the K-based showed an increasing and then decreasing trend. For the mass ratio of alkali activator to metakaolin and Na_2_SiO_3_/MOH, the compressive strength of both geopolymers showed an increasing and then decreasing trend.
(3) For the flexural strength, the increase in the concentration of alkali solution had a positive correlation with the promotion effect on both Na-based and K-based geopolymers. With the increase in the Na_2_SiO_3_/MOH mass ratio, the flexural strength of both site geopolymers showed a trend of increasing and then decreasing. Increasing the mass of the alkali activator negatively affected the flexural strength of the K-based geopolymers, while it showed a trend of increasing and then decreasing for the Na-based geopolymers.
(4) Microscopic analysis showed that increasing the concentration of the alkaline solution could promote the dissolution of silicon–aluminum raw materials and make the reaction more complete. Properly adjusting the ratio of Na_2_SiO_3_ to MOH and the mass ratio of alkali activator to metakaolin could produce more hydrated silicoaluminate gels and C-A-S-H, reduce unreacted metakaolin and microcracks, and cause the structure of the geopolymer to be denser.

## Figures and Tables

**Table 1:** The main chemical compositions (by wt.%) of the MK.

Content	SiO_2_	Al_2_O_3_	CaO	K_2_O	SO_3_	P_2_O_5_	MgO	Na_2_O	Fe_2_O_3_	Total
MK	53.70	35.30	3.88	1.45	1.16	0.40	1.12	1.45	0.25	99.51

**Table 2:** The design of the orthogonal experiment.

Levels	A: Alkaline Solution Concentration (mol/L)	B: Alkali Activator/Binding Material (Mass Ratio)	C: M_2_SiO_3_/MOH (Mass Ratio)
1	8	0.7	1.5
2	10	0.8	2
3	12	0.9	2.5
4	14	1.0	3

**Table 3:** The 7-day and 28-day compressive and flexural strengths of the Na-based geopolymers.

No.	Factors			Compressive Strength (MPa)		Flexural Strength (MPa)	
	A	B	C	7 Days	28 Days	7 Days	28 Days
N1	8 M	0.7	1.5	10.20	26.67	4.75	5.65
N2	8 M	0.8	2	15.27	32.53	4.41	5.81
N3	8 M	0.9	2.5	30.72	36.75	5.21	6.54
N4	8 M	1	3	21.32	34.58	1.10	1.85
N5	10 M	0.7	2	36.66	42.53	5.84	6.46
N6	10 M	0.8	1.5	47.55	59.18	7.03	7.65
N7	10 M	0.9	3	38.42	57.81	6.56	7.29
N8	10 M	1	2.5	22.57	41.14	4.08	4.62
N9	12 M	0.7	2.5	48.96	56.56	6.09	6.72
N10	12 M	0.8	3	49.89	60.16	5.72	6.62
N11	12 M	0.9	1.5	55.25	63.7	5.42	6.17
N12	12 M	1	2	41.40	50.88	5.34	6.05
N13	14 M	0.7	3	45.17	51.73	4.85	5.76
N14	14 M	0.8	2.5	49.48	60.91	7.51	8.10
N15	14 M	0.9	2	57.09	64.18	7.63	8.28
N16	14 M	1	1.5	46.59	56.49	5.78	6.39

**Table 4:** The 7-day and 28-day compressive and flexural strengths of the K-based geopolymers.

No.	Factors			Compressive Strength (MPa)		Flexural Strength (MPa)	
	A	B	C	7 Days	28 Days	7 Days	28 Days
K1	8 M	0.7	1.5	15.09	29.81	5.55	6.65
K2	8 M	0.8	2	15.45	38.41	5.12	6.05
K3	8 M	0.9	2.5	19.69	40.19	5.50	6.4
K4	8 M	1	3	46.10	50.26	1.40	2.13
K5	10 M	0.7	2	46.33	50.50	7.66	8.41
K6	10 M	0.8	1.5	59.11	63.13	7.10	8.09
K7	10 M	0.9	3	49.36	57.20	3.83	4.81
K8	10 M	1	2.5	47.28	53.69	4.27	5.12
K9	12 M	0.7	2.5	62.21	67.70	7.04	7.62
K10	12 M	0.8	3	67.79	72.72	6.98	7.92
K11	12 M	0.9	1.5	58.12	65.53	5.68	6.65
K12	12 M	1	2	49.25	55.42	4.84	6.12
K13	14 M	0.7	3	61.61	65.56	7.98	8.66
K14	14 M	0.8	2.5	60.19	66.11	7.88	8.53
K15	14 M	0.9	2	56.76	64.85	6.14	6.97
K16	14 M	1	1.5	52.18	56.03	3.78	5.24

**Table 5:** Analysis results of the polar differences in the mechanical properties of the Na-based geopolymers at 7 days.

Factors	No.	Compressive Strength (MPa)			Flexural Strength (MPa)		
		A	B	C	A	B	C
K value	K1	77.51	140.99	159.59	15.47	21.53	22.98
	K2	145.20	162.19	150.42	23.51	24.67	23.22
	K3	195.50	181.48	151.73	22.57	24.82	22.89
	K4	198.33	131.88	154.80	25.77	16.30	18.23
	k1	19.38	35.25	39.90	3.87	5.38	5.75
	k2	36.30	40.55	37.61	5.88	6.17	5.81
	k3	48.88	45.37	37.93	5.64	6.21	5.72
	k4	49.58	32.97	38.70	6.44	4.08	4.56
	R	30.21	12.40	2.29	2.58	2.13	1.25
Sequence		A > B > C			A > B > C		
Preferred scheme		A_4_B_3_C_1_			A_4_B_3_C_2_		

**Table 6:** Analysis results of the polar differences in the mechanical properties of the Na-based geopolymers at 28 days.

Factors	No.	Compressive Strength (MPa)			Flexural Strength (MPa)		
		A	B	C	A	B	C
K value	K1	130.53	177.49	206.04	19.85	24.59	25.86
	K2	200.66	212.78	190.12	26.02	28.18	27.03
	K3	231.30	222.44	195.36	25.56	28.71	25.98
	K4	233.31	183.09	204.28	28.96	18.91	21.52
	k1	32.63	44.37	51.51	4.96	6.15	6.47
	k2	50.17	53.20	47.53	6.51	7.05	6.76
	k3	57.83	55.61	48.84	6.39	7.18	6.50
	k4	58.33	45.77	51.07	7.24	4.73	5.38
	R	25.70	11.24	3.98	2.28	2.45	1.38
Sequence		A > B > C			B > A > C		
Preferred scheme		A_4_B_3_C_1_			A_4_B_3_C_2_		

**Table 7:** Analysis results of the polar differences in the mechanical properties of the K-based geopolymers at 7 days.

Factors	No.	Compressive Strength (MPa)			Flexural Strength (MPa)		
		A	B	C	A	B	C
K value	K1	96.33	185.24	184.50	17.57	28.23	22.11
	K2	202.08	202.54	167.79	22.86	27.08	23.76
	K3	237.37	183.93	189.37	24.54	21.15	24.69
	K4	230.74	194.81	224.86	25.78	14.29	20.19
	k1	24.08	46.31	46.13	4.39	7.06	5.53
	k2	50.52	50.64	41.95	5.72	6.77	5.94
	k3	59.34	45.98	47.34	6.14	5.29	6.17
	k4	57.69	48.70	56.22	6.45	3.57	5.05
	R	35.26	4.65	14.27	2.05	3.49	1.13
Sequence		A > C > B			B > A > C		
Preferred scheme		A_3_B_2_C_4_			A_4_B_1_C_3_		

**Table 8:** Analysis results of the polar differences in the mechanical properties of the K-based geopolymers at 28 days.

Factors	No.	Compressive Strength (MPa)			Flexural Strength (MPa)		
		A	B	C	A	B	C
K value	K1	158.67	213.57	214.50	21.23	31.34	26.63
	K2	224.52	241.58	209.18	26.43	30.59	27.55
	K3	262.58	227.77	227.69	28.31	24.83	27.67
	K4	252.55	215.40	246.95	29.4	18.61	23.52
	k1	39.67	53.39	53.63	5.31	7.84	6.66
	k2	56.13	60.40	52.30	6.61	7.65	6.89
	k3	65.65	56.94	56.92	7.08	6.21	6.92
	k4	63.14	53.85	61.74	7.35	4.65	5.88
	R	25.98	7.01	9.44	2.04	3.18	1.04
Sequence		A > C > B			B > A > C		
Preferred scheme		A_3_B_2_C_4_			A_4_B_1_C_3_		

## Data Availability

No new data were created or analyzed in this study. Data sharing is not applicable to this article.
